# Empagliflozin Attenuates Vascular Calcification in Mice with Chronic Kidney Disease by Regulating the NFR2/HO-1 Anti-Inflammatory Pathway through AMPK Activation

**DOI:** 10.3390/ijms241210016

**Published:** 2023-06-12

**Authors:** Chia-Wen Lu, Chung-Jen Lee, Yi-Jen Hsieh, Bang-Gee Hsu

**Affiliations:** 1 Division of Nephrology, Hualien Tzu Chi Hospital, Buddhist Tzu Chi Medical Foundation, Hualien 97002, Taiwan; noirwen@gmail.com (C.-W.L.); hij@mail.tcu.edu.tw (Y.-J.H.); 2 Institute of Medical Sciences, Tzu Chi University, Hualien 97004, Taiwan; 3 Department of Nursing, Tzu Chi University of Science and Technology, Hualien 97005, Taiwan; guggilee@msn.com

**Keywords:** empagliflozin, vascular calcification, AMP-activated protein kinase, nuclear factor erythroid-2-related factor, heme oxygenase 1, chronic kidney disease

## Abstract

Vascular calcification (VC) is associated with increased cardiovascular risks in patients with chronic kidney disease (CKD). Sodium-glucose cotransporter 2 inhibitors, such as empagliflozin, can improve cardiovascular and renal outcomes. We assessed the expression of Runt-related transcription factor 2 (Runx2), interleukin (IL)-1β, IL-6, AMP-activated protein kinase (AMPK), nuclear factor erythroid-2-related factor (Nrf2), and heme oxygenase 1 (HO-1) in inorganic phosphate-induced VC in mouse vascular smooth muscle cells (VSMCs) to investigate the mechanisms underlying empagliflozin’s therapeutic effects. We evaluated biochemical parameters, mean artery pressure (MAP), pulse wave velocity (PWV), transcutaneous glomerular filtration rate (GFR), and histology in an in vivo mouse model with VC induced by an oral high-phosphorus diet following a 5/6 nephrectomy in ApoE^−/−^ mice. Compared to the control group, empagliflozin-treated mice showed significant reductions in blood glucose, MAP, PWV, and calcification, as well as increased calcium and GFR levels. Empagliflozin inhibited osteogenic trans-differentiation by decreasing inflammatory cytokine expression and increasing AMPK, Nrf2, and HO-1 levels. Empagliflozin mitigates high phosphate-induced calcification in mouse VSMCs through the Nrf2/HO-1 anti-inflammatory pathway by activating AMPK. Animal experiments suggested that empagliflozin reduces VC in CKD ApoE^−/−^ mice on a high-phosphate diet.

## 1. Introduction

Chronic kidney disease (CKD) affects nearly 10% of the global population, amounting to >800 million individuals [1]. Patients with CKD display a significant risk for cardiovascular (CV) incidents; half of all individuals with stage 4–5 CKD have CV disease, and CV-related fatalities account for approximately 40–50% of all deaths in patients with advanced CKD and end-stage kidney disease. Over 50% of dialysis patients experience vascular calcification, with a 15–30% yearly rise in coronary calcification, and 40–45% of hemodialysis patients display VC in the aortic region as identified through plain chest radiography [2]. Vascular calcification (VC) is a powerful indicator of CV risk; intimal calcification is related to atherosclerotic plaque formation, whereas medial calcification is characterized by arteriosclerosis [3]. VC is linked to increased CV morbidity and mortality [3,4,5]. Pulse wave velocity (PWV) is a marker of arterial stiffness and is frequently used to assess the risk of CV disease [6]. Risk factors that drive the progression of CV calcification, including hyperphosphatemia, hypercalcemia, and inflammation, are typically present in individuals with CKD and end-stage kidney disease [7]. High levels of serum phosphorus, particularly inorganic phosphate, are a crucial risk factor for VC [8]. Proinflammatory cytokines, such as interleukin (IL)-1β and IL-6, can also contribute to VC by causing the transformation of vascular smooth muscle cells (VSMCs) into osteoblast-like cells, which then help form calcium phosphate crystals within blood vessel walls [9].

Empagliflozin, a sodium–glucose cotransporter 2 (SGLT2) inhibitor, is mainly used to treat type 2 diabetes by inhibiting renal glucose reabsorption. It also has various positive effects on CV and renal health [10]. It activates AMP-activated protein kinase (AMPK) in lipopolysaccharide-treated cardiomyocytes [11]. Empagliflozin improves obesity-related cardiac function by regulating sestrin2-mediated AMPK-mTOR signaling and enhancing the nuclear factor erythroid-2-related factor (Nrf2)–heme oxygenase 1 (HO-1)-mediated oxidative stress response, suggesting antioxidant and anti-inflammatory capabilities [12]. AMPK is an essential cellular energy sensor and a key regulator of metabolic homeostasis. It is also a crucial anti-inflammatory molecule as it suppresses proinflammatory signaling pathways and decreases reactive oxygen species production [13,14]. The precise molecular mechanisms of AMPK activation are still being explored. Further research is required to fully understand these effects and determine the optimal use of empagliflozin for vascular protection.

No therapies have successfully reversed VC in patients with CKD, and only a few have successfully retarded its progression [15,16]. The commonly used five-sixths nephrectomy (5/6 Nx) serves as a standard experimental model for CKD [17,18]. In this study, we aimed to investigate the effects of empagliflozin on a VC model by the surgical approach of a 5/6 Nx mouse model in ApoE-knockout mice (ApoE^−/−^) fed a high-phosphate diet and to identify the possible molecular mechanism of inorganic phosphate-induced VC in VSMCs.

## 2. Results

### 2.1. Empagliflozin Enables the AMPK–Nrf2–HO-1 Pathway to Attenuate Phosphorus-Induced Calcium Deposition in Mouse VSMCs

**Figure 1:**
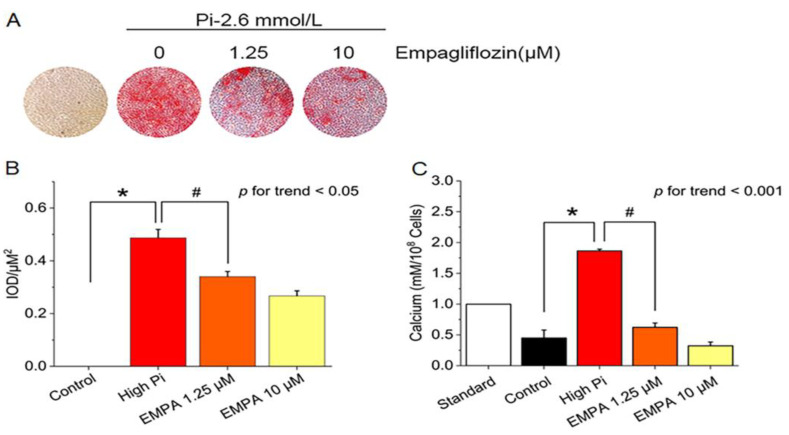
Inhibition of inorganic phosphate (Pi)-induced calcification by empagliflozin. (**A**) Alizarin Red S staining in MOVAS cells. (**B**) Quantification of Alizarin Red S staining positive in MOVAS cells using Image-Pro Plus 6.0 software. (**C**) Intracellular calcium content was determined colorimetrically by using the methylthymol blue method and expressed as millimoles per 10^8^ cells. * *p* for the high-Pi group compared with the control group. # *p* for the empagliflozin 1.25 μM group compared with high-Pi group.

We first examined whether empagliflozin would decrease inorganic phosphate-induced calcium deposition in the mouse VSMCs line (MOVAS cells). The application of varying concentrations of empagliflozin, with or without 2.6 mmol/L inorganic phosphate, resulted in a reduction of calcium deposition in red nodules stained with Alizarin Red S (Figure 1A,B) and intracellular calcium content (Figure 1C).

**Figure 2:**
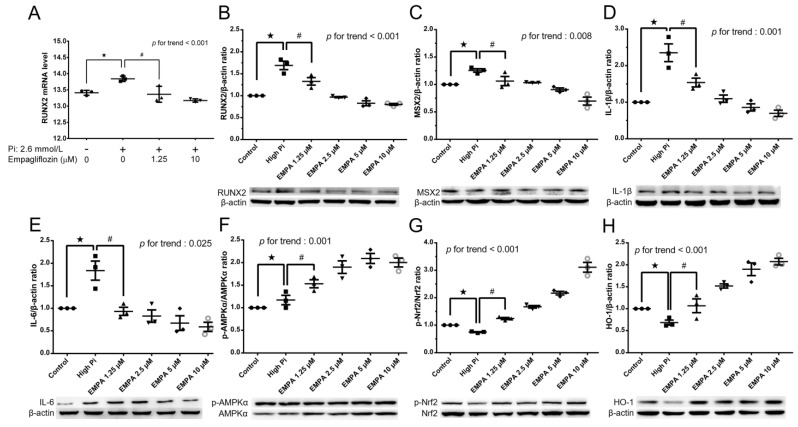
Empagliflozin attenuated high phosphate-induced VC in MOVAS cells through chondro-osteogenic phenotype switching. (**A**) Empagliflozin reduced mRNA abundance of RUNX2 in MOVAS cells treated with 2.6 mM phosphate and/or 1.25–10 μM empagliflozin for 10 days. Immunoblot images and protein expression analysis of (**B**) Runx2, (**C**) MSX2, (**D**) IL-1β, (**E**) IL-6, (**F**) p-AMPK, (**G**) p-Nrf2, and (**H**) HO-1 were represented by empagliflozin in MOVAS cells. Data are presented as mean ± standard error of the mean of three independent experiments performed in triplicates. Results are derived from the mean of triplicate readings from each experiment, and this mean value is utilized for subsequent statistical interpretation and graphical illustration. ★ *p* for the high-Pi group compared with the control group. # *p* for the empagliflozin 1.25 μM group compared with high-Pi group.

After cotreatment of MOVAS cells with high levels of phosphate and empagliflozin, quantitative reverse transcription polymerase chain reaction (qRT-PCR) indicated suppressed runt-related transcription factor 2 (Runx2) mRNA expression (Figure 2A), and Western blotting revealed decreased RUNX2, MSX2, IL-1β, and IL-6 protein levels (Figure 2B–E) and significantly restored phosphorylated AMPK, phosphorylated Nrf2, and HO-1 levels (Figure 2F–H).

**Figure 3:**
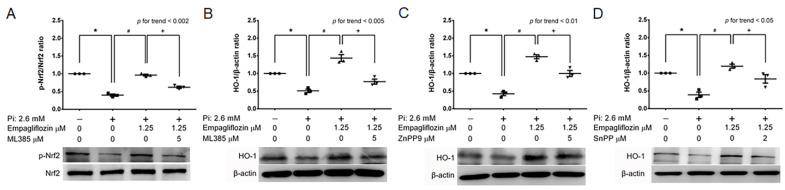
Nrf2 and HO-1 inhibitors counteract the effect of Empagliflozin in suppressing Pi-induced calcification. (**A**,**B**) The Nrf2 inhibitor, ML385 (5 μM), as well as HO-1 inhibitors (**C**) ZnPP9 (5 μM) and (**D**) SnPP (2 μM), were pre-incubated with MOVAS cells for 2 h, followed by an immunoblotting procedure. Data are presented as mean ± standard error of the mean of three independent experiments performed in triplicates. Results are derived from the mean of triplicate readings from each experiment, and this mean value is utilized for subsequent statistical interpretation and graphical illustration. ★ *p* for the high-Pi group compared with the control group. # *p* for the empagliflozin 1.25 μM group compared with high-Pi group. ✦ *p* is used to denote the comparison of groups, with and without inhibitor, in the presence of 1.25 μM empagliflozin.

To further explore if Nrf2 is crucial for the effects mediated by empagliflozin, we attempted to suppress Nrf2′s activity and investigated empagliflozin’s impact on Pi-induced calcification. The introduction of the Nrf2 inhibitor, ML385, led to a noticeable reduction in Nrf2 and HO-1 expression, even with empagliflozin present (Figure 3A,B). The use of HO-1 inhibitors, ZnPP9 and SnPP, counteracted the preventative effect of empagliflozin on Pi-induced calcification (Figure 3C,D). These findings suggest a significant role of the Nrf2/HO-1 signaling pathway in empagliflozin’s protective effect against calcification.

### 2.2. Empagliflozin Reduces Mean Arterial Pressure, PWV in The Mouse Model of VC

**Figure 4:**
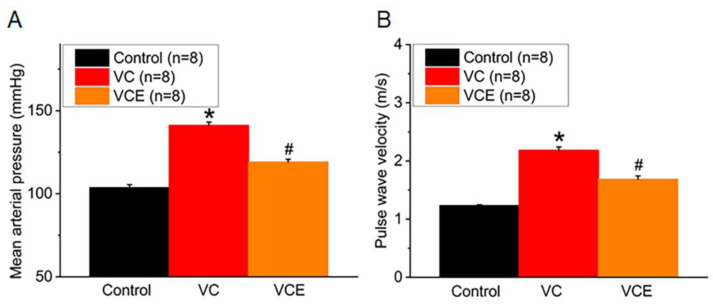
Physiological characteristics of (**A**) mean arterial pressure and (**B**) PWV. * *p* for the VC group compared with the control group. # *p* for the VCE group compared with VC group.

Arterial blood pressure was recorded in conscious mice at the end of Week 16. Mean arterial pressure in the VC group (141.2 ± 1.9 mmHg) was significantly higher than that in the control group (103.7 ± 1.8 mmHg), and empagliflozin treatment in the VC group (VCE; 119.1 ± 1.7 mmHg) decreased the mean arterial pressure significantly compared with the VC group (Figure 4A). PWV was significantly higher in the VC group than in the control group and in the VCE group (Figure 4B). 

### 2.3. Empagliflozin Improves Renal Function in The Mouse Model of VC

**Figure 5:**
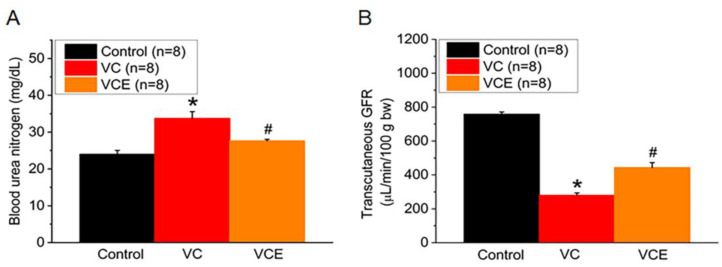
Biomarker of renal function. (**A**) Serum level of blood urea nitrogen; (**B**) transcutaneous GFR. * *p* for the VC group compared with the control group. # *p* for the VCE group compared with VC group.

The VC group exhibited a significant elevation in serum blood urea nitrogen levels when compared to the control group (Figure 5A). The mouse transcutaneous GFR was measured in the conscious state for 90 min; empagliflozin treatment (443.6 ± 32.2 μg/min/100 g bw; VCE group) improved the GFR compared with the VC group (279.9 ± 40.4 μg/min/100 g bw; Figure 5B).

### 2.4. Empagliflozin Improves Serum Level of Calcium, Phosphorus and Decrease Glucose in The Mouse Model of VC

**Figure 6:**
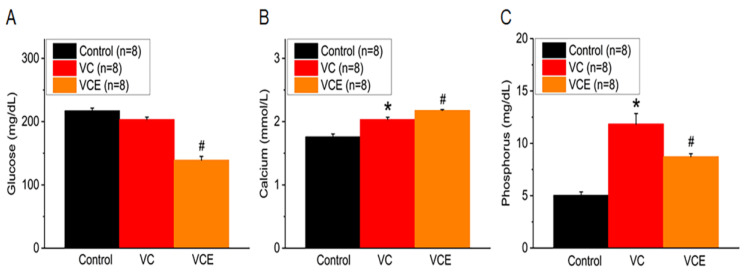
Serum levels of (**A**) glucose, (**B**) calcium, (**C**) phosphorus. * *p* for the VC group compared with the control group. # *p* for the VCE group compared with VC group.

After being fed a high-phosphorus diet for 8 weeks, the blood glucose levels in 5/6 Nx ApoE^−/−^ mice treated with empagliflozin (139.3 ± 5.8 mg/dL) were found to be lower than those in the VC group. Moreover, our findings revealed no significant difference between the control and VC mice (Figure 6A). When compared to the control group, the VC group showed increased serum calcium and phosphorus levels (Figure 6B,C). Empagliflozin treatment (VCE) notably raised serum calcium levels (2.18 ± 0.02 mmol/L; Figure 6B) and reduced phosphorus levels (8.73 ± 0.28 mg/dL; Figure 6C) in comparison to the VC group, suggesting its protective effect against vascular calcification induced by a high-phosphorus diet.

### 2.5. Empagliflozin Decreases Serum Proinflammatory IL-1β, and IL-6 Cytokines in the Mouse Model of VC

**Figure 7:**
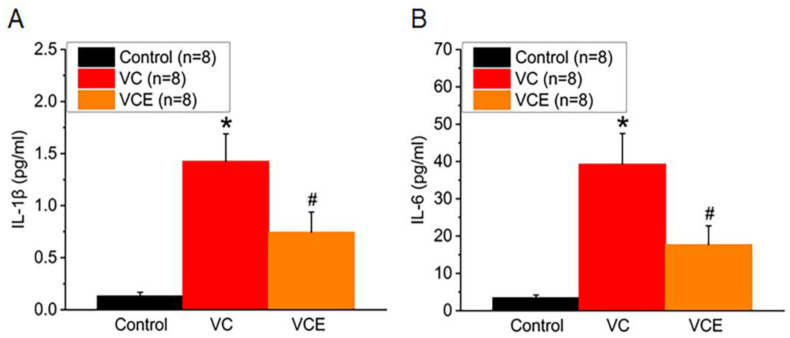
The quantitative measurement of (**A**) IL-1β and (**B**) IL-6 levels in the serum. * *p* for the VC group compared with the control group. # *p* for the VCE group compared with VC group.

We examined the association between inflammatory cytokines and the progression of VC in mice. In comparison to the control group, both VC and VCE groups exhibited significantly higher serum concentrations of IL-1β and IL-6. Empagliflozin effectively lowered the IL-1β increase in the VCE group compared to the VC group (Figure 7A). Furthermore, our results revealed that the VCE group had substantially reduced IL-6 levels relative to the VC group (Figure 7B).

### 2.6. Empagliflozin Decreased Aorta Calcium Deposition in Von Kossa Stain

**Figure 8:**
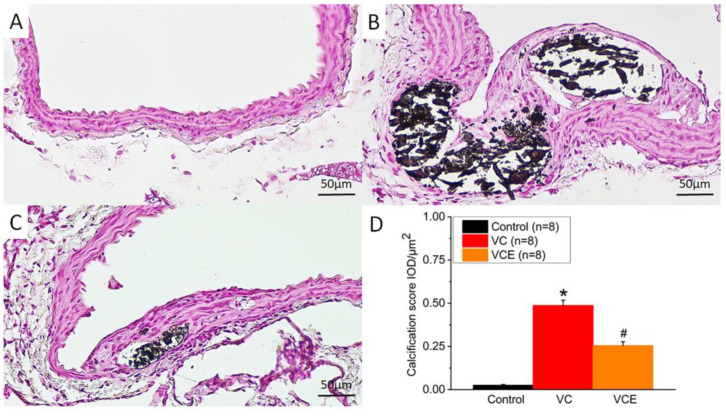
Calcium deposition represented by von Kossa staining in descending thoracic aorta of the (**A**) control, (**B**) VC, and (**C**) VCE groups. (**D**) Quantification of von Kossa staining positive in descending thoracic aorta. * *p* for the VC group compared with the control group. # *p* for the VCE group compared with VC group.

Positive von Kossa staining for calcium phosphate (black or brown-black) was observed within the medial layer of descending thoracic aorta sections in the VC group and VCE group (Figure 8B,C), whereas no calcium deposition was noted in the control group (Figure 8A). We further performed von Kossa stain analysis by using Image-Pro Plus 6.0, with five data points collected from each section (eight sections per group). The quantitative expression level of positive von Kossa staining was determined using integrated optical density–area analysis (Figure 8D). 

### 2.7. Empagliflozin Increased AMPK, αSMA, SM22α Expression in The Mouse Model of VC

**Figure 9:**
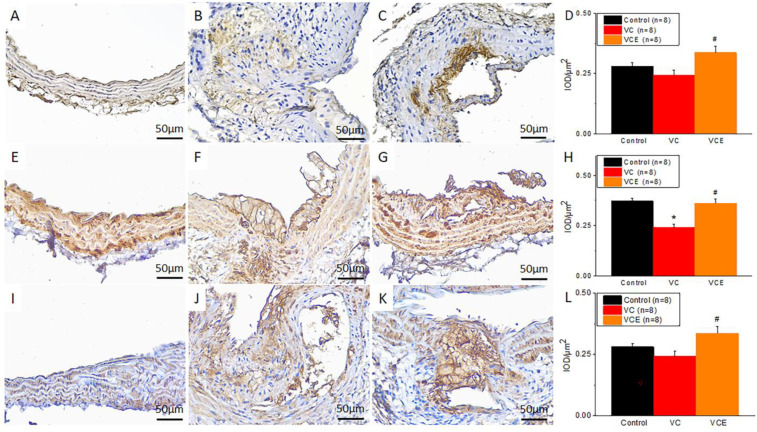
Immunohistochemistry staining of (**A**–**C**) AMPK, (**E**–**G**) αSMA, and (**I**–**K**) SM22α in descending thoracic aorta of the (**A**,**E**,**I**) control, (**B**,**F**,**J**) VC, and (**C**,**G**,**K**) VCE groups. Quantification of (**D**) AMPK, (**H**) αSMA, (**L**) SM22α positive staining in descending thoracic aorta. ★ *p* for the VC group compared with the control group. # *p* for the VCE group compared with VC group.

Our study examined the potential link between the administration of empagliflozin and the activation states of AMPK, alpha-smooth muscle actin (αSMA), and smooth muscle 22α (SM22α) in VC. We performed immunohistochemistry on descending thoracic aorta samples embedded in paraffin to evaluate the levels of AMPK, αSMA, and SM22α. Representative images of these immunohistochemical stains are presented in Figure 9. The resultant brown color corresponds to positive staining. To quantify the level of positive staining for AMPK (Figure 9A–C), αSMA (Figure 9E–G), and SM22α (Figure 9I–K), we utilized integrated optical density-area analysis employing Image-Pro Plus 6.0 software (Figure 9D,H,L). Our data suggest that aortas treated with empagliflozin (VCE group (Figure 9C,G,K)) exhibited greater expression of AMPK, αSMA, and SM22α compared to descending thoracic aortas in the VC group (Figure 9B,F,J).

## 3. Discussion

This study found that empagliflozin attenuates high phosphate-induced calcification of MOVAS cells by regulating the NRF2/HO-1 anti-inflammation pathway through activating AMPK in VSMCs. In addition, animal experiments also confirmed that empagliflozin can decrease VC in 5/6 Nx ApoE^−/−^ mice fed with high-phosphorus diet. 

Patients with CKD, diabetes, and hypertension are prone to VC, particularly in vascular media where VSMCs transdifferentiate into osteogenic cells or undergo apoptosis [19]. Runx2 is crucial for osteoblast differentiation, and low-to-moderate DNA damage can increase Runx2-mediated osteogenic expression [19,20]. Our results indicate that empagliflozin decreased calcium deposition by reducing calcium accumulation in VSMCs. AMPK activation downregulated the expression and activity of Runx2, resulting in the inhibition of osteoblastic differentiation of VSMCs [21]. AMPK mediates the antioxidative cascade by activating Nrf2 [22]. The activation of the Nrf2/HO-1 pathway, mediated by heme, acts as an inhibitor to the calcification of valvular interstitial cells and human lens epithelial cells, and Nrf2 is a regulator of various antioxidant genes and provides a defensive function in valve calcification [23,24]. Our findings reveal that obstructing HO-1 activity with ZnPP9 or SnPP negates the calcification-preventing effects of empagliflozin. Similarly, when ML385 is present, empagliflozin’s capacity to inhibit calcification in MOVAS cells is nullified. Our data indicate that empagliflozin decreased both Runx2 mRNA and protein levels and decreased the expression of proinflammatory IL-6 and IL-1β cytokines by activating AMPK-mediated upregulation of Nrf2-mediated HO-1 expression in VSMCs. In high-fat-diet-fed ApoE^−/−^ mice, empagliflozin was reported to activate AMPK, inhibit atherosclerosis progression, and decreased IL-1β and IL-6 levels [25]. In VC associated with CKD, proinflammatory cytokines such as interleukin-1β (IL-1β) and interleukin-6 (IL-6) may contribute to the transformation of VSMCs into osteoblast-like cells [9]. These inflammatory cytokines can trigger phenotypic alterations in VSMCs, encouraging their conversion into osteoblast-like cells and subsequent mineralization [26]. In our study, we discovered that empagliflozin can enhance AMPK expression and counteract the elevation of serum IL-1β and IL-6 levels triggered by a VC mouse model. This indicates that empagliflozin may offer anti-inflammatory advantages through AMPK activation, leading to reduced calcium deposition in 5/6 Nx ApoE^−/−^ mice subjected to a high-phosphorus diet-induced VC.

VC can result in arterial stiffening and reduced elasticity, thus increasing blood pressure [27,28]. It can also lead to endothelial dysfunction, inflammation, oxidative stress, and other pathophysiological changes, which contribute to hypertension [28]. Several studies have demonstrated a significant correlation between PWV and VC, with higher PWV values associated with higher calcification levels [5,29,30]. This study indicated that empagliflozin can protect against arterial stiffness, as evidenced by a lower PWV, which can decrease blood pressure.

People with CKD, who typically have a low GFR, are at a higher risk of VC than those with normal kidney function [31,32,33]. Decreased GFR is associated with an increased risk of VC, likely due to several factors, including alterations in calcium and phosphate metabolism (such as impaired renal excretion), chronic inflammation, oxidative stress [34], and diminished renal blood circulation, thus promoting kidney disease progression [35]. In CKD patients with an estimated GFR below 30 mL/min/1.73 m^2^, empagliflozin has been shown to decrease the likelihood of kidney disease advancement or cardiovascular-related mortality across a diverse population of individuals at risk for CKD progression [36]. The impaired renal excretion of calcium and phosphate increases their levels in the blood, thus promoting their deposition in the vessel walls. Hyperphosphatemia and hypercalcemia lead to increased calcium and phosphate deposition in blood vessels, triggering RUNX2 expression in VSMCs, which then adopt an osteoblast-like phenotype. αSMA is a marker for adult smooth muscle cells, and its expression decreases following arterial injury [37]. SM22α is crucial for maintaining the smooth muscle cells phenotype [38]. High phosphate levels cause a reduction in SM22α and an increase in Runx2 expression, which intensifies calcification [39]. This exposure to high phosphate levels also induces a shift in cell phenotype from contractile (smooth muscle) to osteo/chondrogenic, marked by decreased expression of αSMA and SM22α, and increased expression of Runx2 [40,41]. Reducing phosphate levels in CKD patients could potentially enhance CV outcomes, as hyperphosphatemia contributes to VC and CV events [42]. However, the evidence for this strategy remains inconclusive. Our findings suggest that empagliflozin treatment may slow down kidney function decline and reduce serum phosphorus levels in 5/6 Nx ApoE^−/−^ mice with VC induced by a high-phosphorus diet, implying that phosphate reduction in CKD could potentially offer beneficial effects in alleviating VC. 

At present, limited research exists examining the connection between empagliflozin and VC. Medial calcification, which refers to calcification in the smooth muscle layer of the arterial wall, is particularly prevalent among patients with CKD [43]. Our findings from a pathological analysis reveal a notable decrease in calcium deposits within the tunica media following empagliflozin treatment in CKD ApoE^−/−^ mice subjected to a high-phosphorus-diet-induced VC. As a result, empagliflozin could potentially be used to treat VC, although further evidence is required to substantiate this hypothesis.

## 4. Materials and Methods

### 4.1. Antibodies and Chemicals

Antibodies against the MSX2, AMPKα, Phospho-AMPKα, β-actin, and GAPDH homeobox protein were purchased from Cell Signaling (Cell Signaling Technology, MA, USA). Antibodies against NRF2, Phospho-NRF2, HO-1, IL-6, and IL-1β were purchased from ABclonal (ABclonal, Woburn, MA, USA). Antibodies against Runx2 were purchased from Abcam (Abcam, Cambridge, UK). Methylthymol blue tetrasodium salt was purchased from Thermo Fisher Scientific/ACROS organics (Acros Organicas, Geel, Belgium). The Nrf2 inhibitor, ML385 (GC19254), was purchased from GLPBIO (Montclair, CA, USA). The HO-1 inhibitors, Zinc protoporphyrin-9 (ZnPP9, SC-200329) and Sn-protoporphyrin IX (SnPP, SC-203452), were procured from Santa Cruz Biotechnology (Dallas, TX, USA). Empagliflozin was obtained from AdipoGen Life Sciences (AdipoGen Life Sciences, San Diego, CA, USA). Alizarin Red S was purchased from PanReac Applichem (PanReac Applichem, Monza, Italy). High-glucose Dulbecco modified Eagle’s medium (DMEM), fetal bovine serum, and G418 antibiotics were purchased from Invitrogen (Invitrogen, Carisbad, CA, USA). The Bio-Rad protein assay reagent was purchased from (Bio-Rad Laboratories, Hertfordshire, UK). Sodium phosphate monobasic and other reagents were purchased from Sigma-Aldrich (Sigma-Aldrich, St. Louis, MO, USA). All chemicals were prepared and stored in accordance with the manufacturers’ recommendations.

### 4.2. MOVAS Cell Culture

The MOVAS cells (ATCC; Manassas, VA, USA; CRL-2797), a mouse VSMCs line, were cultured in high-glucose DMEM supplemented with 10% fetal bovine serum (Life Technologies Inc., Gaithersburg, MD, USA) and 0.2 mg/mL G418 antibiotics (Sigma-Aldrich, St. Louis, MO, USA). Incubation was conducted at 37 °C in a humidified atmosphere with 5% CO_2_, and the cells were subcultured every second day in the designed medium.

### 4.3. Induction of Calcification

VSMCs were cultured in DMEM growth medium and subcultured at 80% confluence. The growth medium was supplemented with (calcification medium) or without (normal medium) 2.6 mM sodium phosphate monobasic for another 10 days to induce calcification. During this time, the cells were subcultured every 2 days. For time course experiments, the first day of culture in the calcification medium was defined as day 1.

### 4.4. Alizarin Red S Staining

To determine calcification status, Alizarin Red S staining was performed after a 10-day incubation with normal-phosphate or high-phosphate medium and various empagliflozin concentrations. The cells were fixed with 95% ethanol for 30 min at room temperature and then stained with Alizarin Red S (0.1%, pH 4.3) at 37 °C in a humidified, 5% CO_2_ atmosphere. The calcified areas were stained red under a light microscope.

### 4.5. Quantification of Calcium Content

After the indicated incubation period, MOVAS cells were decalcified with 0.6 mol/L HCl at 37 °C overnight, and calcium content in the supernatant was determined colorimetrically by using the methylthymol blue method in accordance with the manufacturer’s protocol. The amount of free calcium was expressed as millimoles per 10^8^ cells.

### 4.6. qRT-PCR Analysis

We assessed the expression of the osteogenic gene Runx2 by employing qRT-PCR. Total RNA was extracted from MOVAS cells using Trizol (Ambion, Thermo Fisher Scientific, Waltham, MA, USA) reagent, following the standard isolation protocol. RNA concentration was determined using a NanoDrop spectrophotometer (Thermo Fisher Scientific). We used 1 µg of RNA to synthesize complementary DNA (cDNA) with the iScript cDNA Synthesis Kit (Bio-Rad Laboratories, Hercules, CA, USA). A mixture of 2 µL cDNA, 10 µL SYBR Green (Thermo Fisher Scientific), 0.5 µL each of forward and reverse primers, and 7 µL ddH_2_O was prepared and quantified using a real-time reverse transcription linkage instrument (StepOnePlus Real-Time PCR System, Thermo Fisher Scientific). The experiment was performed three times. Data were normalized using GAPDH as an internal control and relative mRNA expression change between 2 groups was calculated by 2^−ΔΔCt^ method [44].

### 4.7. Western Blotting

For each of three independent experiments, total protein from MOVAS cells was extracted after treatment with RIPA lysis buffer supplemented with complete protease and a phosphatase inhibitor cocktail. After centrifugation at 12,000× *g* for 30 min, the protein content of the cells was measured using the Bio-Rad protein assay reagent (Bio-Rad Laboratories, Hertfordshire, UK), with BSA as the standard. After protein denaturation, equal amounts of proteins were separated on SDS-PAGE and transferred to PVDF membranes, which were then blocked with 5% nonfat milk. The membranes were incubated overnight at 4 °C with primary antibodies—i.e., antibodies against RUNX2, MSX-2, GAPDH, and β-actin, AMPKα, phospho-AMPKα, NRF2, phospho-NRF2, HO-1, ML385, Znpp-9, SnPP, IL-6, IL-1β—followed by the addition of appropriate horseradish peroxidase-conjugated secondary antibodies and detected using ECL (GE). The chemiluminescent signal was detected using a UVP BioSpectrum 810 imaging system (Thermo Fisher Scientific). Bands were quantified with ImageJ software 1.52a.

### 4.8. Animals

**Figure 10:**
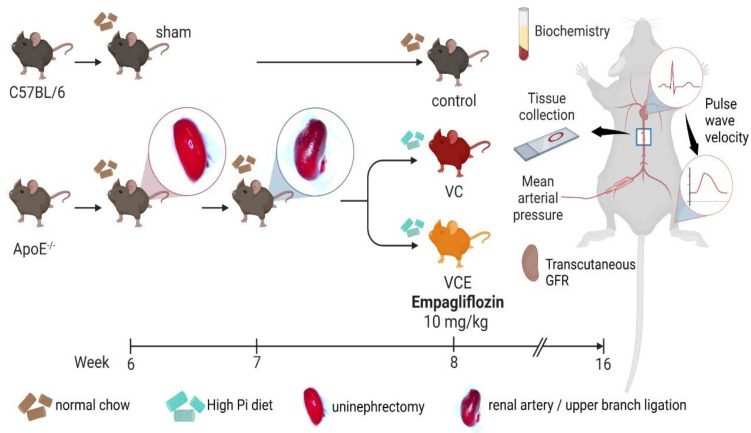
Schematic of animal experiment. Created with BioRender.com.

Eight male C57BL6 mice (20–25 g) were purchased from BioLASCO (Taipei, Taiwan), and 16 male Apoe^tm1Unc^/J mice (20–25 g) were obtained from Jackson Laboratory (Bar Harbor, ME, USA). The 24 mice were divided into three equal groups (8 per group): C57BL/6 of sham fed normal chow (the control group), ApoE^−/−^ of 5/6 nephrectomy (5/6 Nx) fed a high-phosphorus diet (the VC group), ApoE^−/−^ of 5/6 nephrectomy fed both a high-phosphorus diet and empagliflozin (the VCE group; Figure 10). The VCE group received oral empagliflozin 10 mg/kg for 8 weeks after 5/6 nephrectomy. All mice were housed at the Laboratory Animal Center of Tzu Chi University (Hualien, Taiwan). The experimental protocols were approved by the Institutional Animal Care and Use Committee of Hualien Tzu Chi Hospital (approval number 108-48).

### 4.9. Animal Model of VC

The VC mouse model was established by subjecting them to 5/6 Nx with a high-phosphorus diet as described elsewhere [18]. We modified 5/6 Nx into a two-stage procedure [17,18]. The mice were anesthetized with isoflurane inhalation (Forane, Baxter, Deerfield, IL, USA) by using a vaporizer (Matrx VIP 3000, Midmark, Dayton, OH, USA). Under anesthesia, the right kidney was removed in the first stage, and the upper branch of the left renal artery was ligated using a 6-0 catgut absorbable suture 7 days later. Following 5/6 Nx, the VC mice were fed a high-phosphorus (1.5% total phosphorus) diet (Product #D09051102, Research Diets, New Brunswick, NJ, USA) for 8 weeks.

### 4.10. Mean Arterial Pressure

The mice were anesthetized using isoflurane as described earlier. During anesthesia, the femoral artery was cannulated and connected to a pressure transducer to record mean arterial pressure on a polygraph recorder (e-corder 410, eDAQ, Denistone East, Australia). The operation was completed in 15 min, leaving a small section wound (<0.5 cm^2^). The mice soon awakened, and arterial blood pressure was recorded in the conscious mice [45].

### 4.11. Pulse Wave Velocity 

PWV was acquired from the cardiovascular pulse, distance between locations *d*, and transit time Δ*t* for the pulse to travel distance *d*. After isoflurane anesthesia, the mice were placed supine, and distance *d* between the supraclavicular notch and the ankle of the left hindlimb was measured. Three acupuncture needle electrodes were inserted subcutaneously into the right and left forelimbs and the left hindlimb. The other ends of the electrodes were connected to an amplifier cable for electrocardiography (ECG) of lead II (Powerlab/8sp, BIO Amp, ADinstruments, Dunedin, New Zealand). The pulse oximeter (MouseOx, Starr Life Sciences, Oakmont, PA, USA) was placed on the left ankle. The transit time (Δ*t*) was acquired using the pulse wave between the initial peak of the ECG R-wave and arrival peak of pulse oximeter wave (LabChart software v7, Adinstruments, New Zealand). PWV was calculated as follows: PWV = *d* (meters)/Δ*t* (seconds).

### 4.12. Transcutaneous GFR

Transdermal GFR in mice was measured using the excretion kinetics of fluorescein-isothiocyanate conjugated sinistrin (FITC-sinistrin) [45]. After hair was removed and the transdermal GFR Monitor (MediBeacon, Louis, MO, USA) was attached, the mice were anesthetized with isoflurane as described earlier and administered FITC-sinistrin (Mannheim Pharma and Diagnostics, Mannheim, Germany), 0.15 mg per gram body weight (g bw), through the retro-orbital venous sinus. The duration for transdermal GFR measurement was 1.5 hours. The GFR was calculated from the measured half-life of FITC-sinistrin clearance using software (MPDStudio Version RC15, MediBeacon, Louis, MO, USA).

### 4.13. Tissue Collection, Biochemical Analysis, and Serum Cytokines Analysis

After the animals were sacrificed, blood samples were collected and centrifuged at 10,000× *g* for 10 min at 4 °C to separate the serum. Serum biochemical measurements of glucose, blood urea nitrogen, calcium, and phosphorus were recorded using a biochemistry analyzer (Spotchem SP-4430, Arkray, Minneapolis, MN, USA) [46]. The remaining serum was preserved at −80 °C prior to evaluation of IL-1β and IL-6 using an enzyme-linked immunosorbent assay kit and commercial assay kits (R&D Systems, Minneapolis, MN, USA) [46]. The tissue specimens were fixed in 4% buffered formaldehyde and then subjected to von Kossa staining.

### 4.14. Von Kossa Staining

Sections of the descending thoracic aorta were deparaffinized, rehydrated, and incubated in silver nitrate solution (5%) for 60 min with 100-watt incandescent. The sections were incubated in sodium thiosulfate (5%) solution (5%) for 2 min and nuclear fast red solution for 5 min and rinsed in distilled water before and after incubation. The slides were dehydrated, mounted, and observed under light microscopy. Von Kossa stain was evaluated on the basis of the average optical density of positive reactions using Image-Pro Plus 6.0 software [47].

### 4.15. Immunohistochamistry Staining

Descending thoracic aorta sections, 3 μm thick, were deparaffinized, rehydrated, and underwent microwave-assisted antigen retrieval using Trilogy (Cell Marque, Rocklin, CA, USA). Following this, the sections were treated to block endogenous peroxidase activity with a 3% hydrogen peroxide solution for 5 min and a 10% bovine serum albumin-containing phosphate-buffered saline for 30 min at room temperature. The primary antibodies used were AMPK (1:150 dilution; Cell Signaling Technology, Danvers, MA, USA), α-SMA (1:200, ab124964, Abcam), and SM22α (1: 200 dilutions, ab155272, Abcam), for 30 min. After washing three times, biotinylated mouse anti-mouse secondary antibodies were applied to the sections for 30 min at room temperature. The reaction was visualized using 3,3′-diaminobenzidine, counterstained with Mayer’s hematoxylin, and dehydrated with ethanol before being coverslipped for assessment. Light microscopy was used to observe the slides, and the average optical density of positive reactions was determined for immunohistochemical analysis using Image-Pro Plus 6.0 software [47].

### 4.16. Statistical Analysis

Data are presented as the mean ± standard error of the mean. For multiple comparisons, significance was assessed using a one-way analysis of variance with Bonferroni’s post hoc test or the Cochran–Armitage test for trends. The Statistical Package for the Social Sciences (SPSS) (version 19.0; SPSS Inc., Chicago, IL, USA) was used for statistical analysis. Groups were compared using an unpaired t test. *p* < 0.05 was considered statistically significant. 

## 5. Conclusions

VC is a complex process driven by numerous factors that lead to calcium phosphate accumulation in arterial walls, and the consequent arteriosclerosis increases blood pressure. Our results indicate that empagliflozin reduces high phosphate-induced calcification in MOVAS cells by activating the AMPK–Nrf2–HO-1 pathway and decreases VC in the descending thoracic aortas of ApoE^−/−^ mice subjected to 5/6 Nx and fed a high-phosphorus diet. 

In addition to these findings, our study also revealed that empagliflozin suppresses the expression of osteogenic markers, such as Runx2, in MOVAS cells. This suggests a potential mechanism by which empagliflozin may inhibit the trans-differentiation of VSMCs into osteoblast-like cells, a critical process in the development of VC. Furthermore, our in vivo experiments indicate that empagliflozin treatment can improve renal function and reduce serum phosphate levels in the 5/6 Nx ApoE^−/−^ mice model, which could contribute to the observed decrease in VC.

In conclusion, our findings not only highlight the potential role of empagliflozin in reducing high-phosphate-induced calcification in MOVAS cells but also propose its potential therapeutic application in alleviating VC in the context of CKD and other related conditions. Future studies may further elucidate the precise molecular mechanisms and explore the clinical implications of these results.

## 6. Limitations

Empagliflozin is administered at 10 mg once daily in patients with CKD [36] and type 2 diabetes mellitus [48]. In our animal study, we used a dosage of 10 mg/kg of empagliflozin in a mouse model for VC. However, the potential therapeutic effect and the appropriate clinical dosage of empagliflozin for VC still necessitate further elucidation and investigation. We recognize that continued research is essential to bridge the gap between the findings from animal studies and the outcomes of clinical applications.

## Data Availability

The data that support the findings of this study are available on request from the corresponding author.
